# SARS‐CoV‐2 Seroprevalence Trends in the Netherlands in the Variant of Concern Era: Input for Future Response

**DOI:** 10.1111/irv.13312

**Published:** 2024-06-04

**Authors:** Eric R. A. Vos, Cheyenne C. E. van Hagen, Denise Wong, Gaby Smits, Marjan Kuijer, Alienke J. Wijmenga‐Monsuur, Joanna Kaczorowska, Robert S. van Binnendijk, Fiona R. M. van der Klis, Gerco den Hartog, Hester E. de Melker

**Affiliations:** ^1^ Centre for Epidemiology and Surveillance, Centre for Infectious Disease Control National Institute for Public Health and the Environment (RIVM) Bilthoven The Netherlands; ^2^ Centre for Immunology of Infectious Diseases and Vaccines, Centre for Infectious Disease Control National Institute for Public Health and the Environment (RIVM) Bilthoven The Netherlands; ^3^ Laboratory of Medical Immunology, Radboud Institute for Molecular Life Sciences Radboudumc Nijmegen The Netherlands

**Keywords:** breakthrough infections, COVID‐19, pandemic preparedness, prospective cohort, risk groups, SARS‐CoV‐2, seroprevalence trends, the Netherlands

## Abstract

**Background:**

To inform future response planning we aimed to assess SARS‐CoV‐2 trends in infection‐ and/or vaccine‐induced immunity, including breakthrough infections, among (sub)groups, professions and regions in the Dutch population during the Variant of Concern (VOC)‐era.

**Methods:**

In this prospective population‐based cohort, randomly selected participants (*n* = 9985) aged 1–92 years (recruited early‐2020) donated home‐collected fingerstick‐blood samples at six timepoints in 2021/2022, covering waves dominated by Alpha, Delta, and multiple Omicron (sub‐)variants. IgG antibody assessment against Spike‐S1 and Nucleoprotein was combined with vaccination‐ and testing data to estimate infection‐induced (inf) and total (infection‐ and vaccination‐induced) seroprevalence.

**Results:**

Nationwide inf‐seroprevalence rose modestly from 12% (95% CI 11–13) since Alpha to 26% (95% CI 24–28) amidst Delta, while total seroprevalence increased rapidly to 87% (95% CI 85–88), particularly in elderly and those with comorbidities (i.e., vulnerable groups). Interestingly, highest infection rates were noticeable among low/middle educated elderly, non‐Western, those in contact professions, adolescents and young adults, and in low‐vaccination coverage regions. Following Omicron emergence, inf‐seroprevalence elevated sharply to 62% (95% CI 59–65) and further to 86% (95% CI 83–90) in late‐2022, with frequent breakthrough infections and decreasing seroprevalence dissimilarities between most groups. Whereas > 90% of < 60‐year‐olds had been infected at least once, 30% of vaccinated vulnerable individuals had still not acquired hybrid immunity.

**Conclusions:**

Groups identified to have been infected disproportionally during the acute phase of the pandemic require specific attention in evaluation of control measures and future response planning worldwide. Furthermore, ongoing tailored vaccination efforts and (sero‐)monitoring of vulnerable groups may remain important.

## 1 Introduction

**FIGURE 1:**
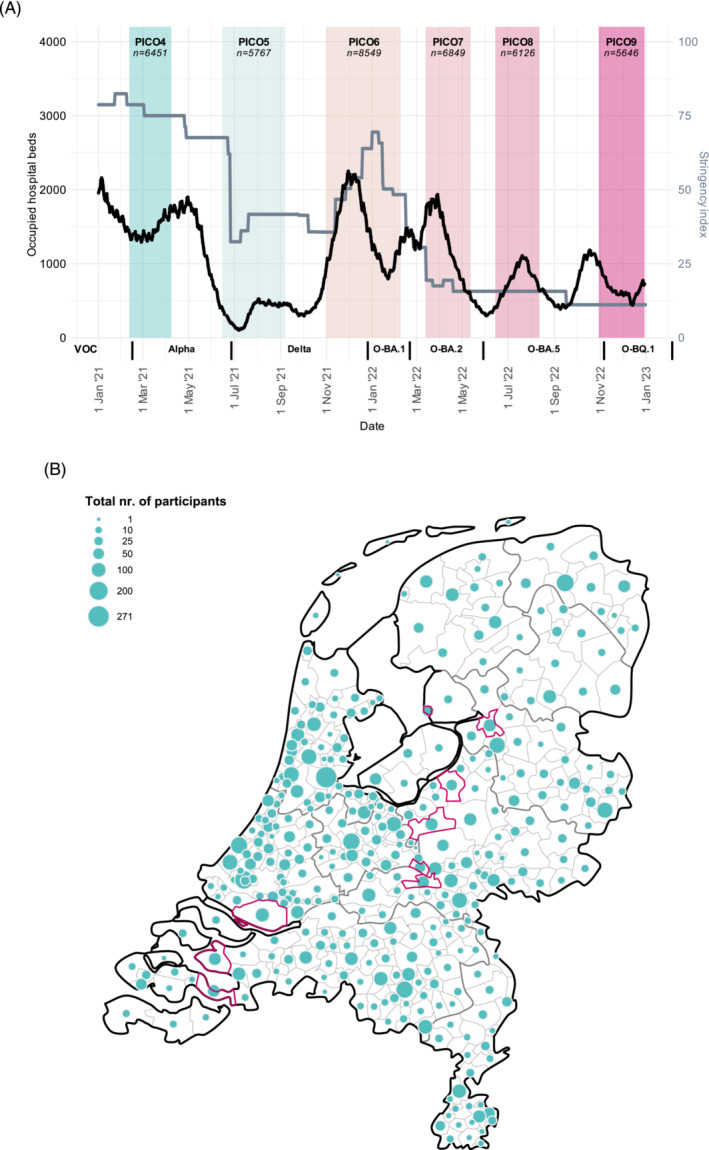
(A) The PIENTER Corona (PICO) study timeline in 2021 and 2022, by daily occupied hospital beds due to COVID‐19 (black line, left y‐axis), daily stringency index (gray line, right y‐axis) and dominance of specific Variants of Concern (VOC) in the Netherlands (on x‐axis, from left to right: Alpha, Delta, Omicron‐BA.1, ‐BA.2, ‐BA.5, and ‐BQ.1). A nationwide vaccination campaign started in the beginning of January 2021 and followed an age‐, comorbidity‐ and healthcare worker‐based prioritization. The number of participants analyzed in the current study are provided per study round and depicted in italic in the colored boxes. Length of the study rounds are consistent with the width of the colored boxes, although the majority of participants participated at the beginning of each study round, also reflected by the median and interquartile ranges (IQR): PICO4: 17 (15–19) February 2021; PICO5: 23 (21–28) June 2021; PICO6: 11 (8–15) November 2021; PICO7: 23 (19–28) March 2022; PICO8: 21 (19–27) June 2022; and PICO9: 9 (5–14) November 2022. Data on hospital bed occupation are open source and were downloaded from the National Coordination Center for Patient Distribution (LCPS) (via: https://lcps.nu/datafeed). Stringency index data, here depicted for the Netherlands, are open source too and were downloaded from Our World in Data (via: https://ourworldindata.org/covid‐stringency‐index. (B) Nationwide distribution of the total number of unique PICO participants analyzed (*n* = 9985), by municipality. The current study comprises the national sample (*n* = 9492) and the low vaccination coverage (LVC) sample (*n* = 493). The number (proportional to the size of the green dots) and distribution of participants match the countrywide population size and ‐distribution. Pink‐circled municipalities represent the LVC municipalities (as per the PIENTER‐3 cohort). Thicker gray lines represent the borders of the provinces and the thin gray lines those of the municipalities.

Severe acute respiratory syndrome coronavirus‐2 (SARS‐CoV‐2), causative agent of coronavirus disease 2019 (COVID‐19), has imposed a tremendous burden on societies worldwide since its origin in late 2019. In the Netherlands, stringent measures including social distancing and several lockdowns (with fluctuating stringency; Figure 1A) have been initiated to curb transmission and prevent the health system from collapsing. Nonetheless, initial waves of infection in spring 2020 and later in winter caused huge spikes in hospitalizations and over 20,000 deaths [1]. Although some regions were hit relatively hard, only a small proportion of the population had been infected, namely, 2.8% at the peak of the first wave in April 2020 and 4.5% after the first wave in June 2020, as estimated from our earlier nationwide seroepidemiological investigations [2, 3].

A nationwide vaccination campaign started in January 2021 and followed an age‐, comorbidity‐ and healthcare worker (HCW)‐based prioritization [4]. Vaccination coverage was generally high and effectively changed the epidemiology, yet at the same time Variants of Concern (VOC) emerged. The increased transmissibility [5] and severity of Alpha and, thereafter, Delta caused a steep rise in reported cases, hospital‐ and intensive care unit (ICU) admissions and deaths predominantly among unvaccinated individuals [5, 6, 7, 8, 9, 10], thus mainly affecting groups with low access and uptake of vaccines [11]. All persons aged ≥ 12 years had been offered primary vaccination before the end of 2021. The increasing number of reports of waning vaccine effectiveness triggered administration of booster doses in autumn 2021 [12], (firstly) to vulnerable groups and HCW, and later to everyone ≥ 18 years during winter [4]. Signs of enhanced immune escape became even more apparent with emergence of Omicron in late 2021, reflected by high numbers of reported (breakthrough) cases. Relative fewer persons became severely‐ill, enabling relaxation of control measures in spring 2022 [10, 13]. Since the beginning of 2022, primary vaccination has been available for 5‐ to 11‐year‐olds, yet nationwide coverage has been low (2%) [10]. Second boosters, targeted to at‐risk groups, were offered in spring of 2022, and bivalent boosters followed that autumn [14].

To assist public health decision‐making during different phases of the pandemic, for example, regarding effectiveness of control measures and vaccination, or insights on severity of disease, it is essential to understand population dynamics of infection and vaccination, and potential disparities between groups [3, 8]. However, traditional surveillance methods underestimate prevalence and incidence as they rely on testing policy and (self/home) testing behavior, or because a proportion of cases is asymptomatic and remains undiagnosed [15]. Hence, seroepidemiology provides a unique opportunity in complementing other surveillance tools by estimating the prevalence of antibodies induced by infection and/or vaccination, generally indicative of protection against (severe) disease. However, the majority of serosurveys have a number of limitations, for instance: (1) usage of convenience samples that lack representativeness of the general population and in‐depth questionnaire data, such as sample sets consisting of only healthcare workers or blood donors, or not including children and elderly; (2) usage of cross‐sectional sample sets which carry a risk of missing previous infections due to waning immunity; or (3) sole qualitative assessment of the presence of antibodies, which limits the detection of re‐infections [16].

Therefore, by means of the large nationwide population‐based prospective PIENTER Corona (PICO) serosurveillance study, we seromonitored the Dutch population repeatedly across all ages throughout the acute phase of the pandemic at multiple timepoints in order to inform (short‐term) decision‐making. Here, by means of this prospective cohort, we aim to assess trends in infection‐ and/or vaccine‐induced immunity, including breakthrough infections, among different (sub)groups and regions in the population during the VOC‐era in 2021–2022. These findings can serve as input for evaluation of the pandemic response and construction of future preparedness globally, and guide ongoing preventive strategies in the endemic/post‐pandemic period.

## 2 Methods

### 2.1 Study Settings

Participants were recruited from the Dutch population registry via random selection (without exclusion criteria) in a region‐ and age‐specific (covering 1–90 years) manner. Details on the sampling at the set‐up of this prospective PICO‐cohort (using the framework of the PIENTER3 serosurvey established in 2016/2017 [17]) and the additional sampling prior to round 2 (PICO2, June 2020, after the first wave) have been described comprehensively [2, 3]. The PICO‐cohort consists of a national sample with participants from across the country resembling the Dutch population (NL); and a sample with participants from municipalities with a lower childhood vaccination coverage (LVC, conceived in PIENTER3), inhabited by a relative large portion of Orthodox‐Reformed Protestants who largely refuse vaccination on religious grounds (commonly referred to as “the Bible belt”). Prior to PICO6 (November 2021), NL was supplemented similar to previous sampling with randomly‐selected persons (predominately younger and older age groups due to drop‐outs) to maintain power. Details are provided in the supporting information and Table S1.

The PICO‐study protocol was approved by the Medical Ethics Committee MEC‐U, the Netherlands (Clinical Trial Registration NTR8473), conformed to the principles embodied in the Declaration of Helsinki, and all participants (or parents/guardians) provided written informed consent.

### 2.2 Data Collection

In the current study, we describe six follow‐up timepoints during the VOC‐era (2021–2022), covering PICO4–9—and used data from prior rounds where applicable. Median inclusion date in PICO4 (17 February 2021) matched the start of Alpha dominance (i.e., PICO4 findings display the pre‐Alpha period). Intervals between median dates of subsequent rounds greatly match waves dominated by single VOCs (except for Delta that covered PICO6 and also partly PICO7 due to its dominance up until the end of December 2021), namely: Alpha (PICO5: 23 June 2021), Delta (PICO6: 11 November 2021), Omicron‐BA.1 (PICO7: 23 March 2022), Omicron‐BA.2 (PICO8: 21 June 2022), and Omicron‐BA.5 (PICO9: 9 November 2022) (Figure 1A). Each round, fingerstick blood samples were home‐collected using contact‐activated lancets (BD microtainer) and mini‐collect serum tubes (Greiner), which were returned to the RIVM‐laboratory in safety envelopes. Serum was processed and stored at −20°C awaiting analyses. Concurrently, participants were requested to complete a(n) (online) questionnaire regarding sociodemographics, occupation, comorbidities, self‐reported SARS‐CoV‐2 testing (PCR/rapid‐antigen test) and vaccination data, and factors related to potential exposure.

### 2.3 Serological Analyses

Serum samples were quantitatively analyzed for SARS‐CoV‐2 immunoglobulin G (IgG) antibodies against Spike‐S1 and Nucleoprotein (N) antigens using a validated fluorescent bead‐based multiplex‐immunoassay, as described previously [18]. Briefly, samples were diluted and incubated with S1‐ (Sino Biological, 40591‐V08H) and N‐ (Sino Biological, 40588‐V08B) coupled beads in SM01‐buffer (Surmodics, USA) supplemented with 2% FCS, while shaking at room temperature in the dark for 45 min. Plates were washed three times with PBS, and incubated with PE‐conjugated goat anti‐human IgG (Jackson ImmunoResearch, 109‐116‐098) for 30 min. Following final washing steps, samples were acquired on a Luminex FlexMap3D. Antibody concentrations were interpolated from pooled sera calibrated against the World Health Organization (WHO) standard (NIBSC, 20/136) using a 5‐parameter logistic fit. Seropositivity for anti‐S1 was considered at a cut‐off concentration of 10 binding antibody units (BAU) per milliliter (mL) and for anti‐N at 14.3 BAU/mL, as derived from previous mixture modeling and receiver operator characteristics analyses [3, 19]. Applying these cut‐offs to detect infection resulted in a specificity for anti‐S1 of 99.9% and a sensitivity of 94.3%, and for anti‐N of 98.0% and 86.0%, respectively.

### 2.4 Statistical Analyses

Data cleaning, management, and analyses were performed in SAS v.9.4. (SAS Institute Inc., USA) and R v.4.2.2.

#### 2.4.1 Definitions

At each study round, serological information (since the start of the PICO‐study) was combined with vaccination‐ and testing data to assess infection‐induced (inf) (at least once) and total (i.e., infection‐ and vaccination‐induced, equivalent to S1‐seropositivity) seroprevalence. More specifically, inf‐seroprevalence was determined via anti‐S1 seropositivity in unvaccinated (who could potentially serorevert); and in vaccinated individuals via anti‐N seropositivity, a SARS‐CoV‐2 positive test, or a ≥ 4‐fold‐increase in anti‐S1 given they had not received a vaccine dose 4 weeks before their previous collection (i.e., the time for a vaccine response to peak, thereby ruling‐out a potential further increase since previous collection due to vaccination and thus fold‐increase in the current round). Participants aged ≥ 12 years enrolled since PICO6 were excluded for these infection analyses as they lacked serological history from previous rounds. Since anti‐N wanes relatively fast—and was the preferred marker we had to rely on since most were vaccinated at that time—this would have underestimated previous infections (particularly those from the beginning of the pandemic) [19]. Moreover, breakthrough infections could be identified in vaccinated persons who presented a positive SARS‐CoV‐2 test (at least 14 days) after their first vaccination, seroconverted for anti‐N in rounds after their first vaccination or had a ≥ 4‐fold‐increase if already anti‐N seropositive, or had a ≥ 4‐fold‐increase in anti‐S1 given they had not received a vaccine dose 4 weeks before their previous collection.

#### 2.4.2 Analyses

Whole population (overall) and (sub)groups seroprevalence estimates (total and inf), vaccination coverage and ‐doses, and proportion of breakthrough infections (and their 95% confidence intervals (CIs)) were calculated. The survey design was taking into account and weights (per study sample) were incorporated using a set of sociodemographic characteristics (age, sex, ethnic background and urbanization degree) to match the Dutch population distribution following census data (of 1 January 2020) from the Statistics Netherlands. Infection estimates were controlled for test performance characteristics subsequently [20]. Differences in seroprevalence between (sub)groups were determined by estimating the parameters of the beta distribution for these estimates using the methods of moments [21]. Risk ratios, their corresponding 95% CIs and *p* values were estimated by Monte Carlo simulations of the seroprevalence estimates; *p* values were reported where applicable, and those < 0.05 were considered statistically significant. Smooth age‐specific seroprevalence estimates were modeled with surveylogistic regressions incorporating B‐splines, using Akaike's information criteria (AIC) for optimization of the number of percentile‐placed knots.

## 3 Results

### 3.1 Study Population

Table 1 shows the sociodemographic characteristics, comorbidity‐ and vaccination status of participants throughout the study period. The total number of unique participants with a serological assessment in the current cohort was 9985 and fluctuated between 5767–8549 across study rounds; of which NL comprised 5371–8138 and LVC 275–470. Drop‐outs ranged between 8% and 20% per study round, yet the proportions of all sociodemographics groups were comparable over time. In NL, age ranged between 1 and 92 years, with a median between 51–57 years, and generally highest participation in adults up to 80 years. The five sampled regions were consistently similarly (~20%) distributed across the cohort and over time (Figures S1 and 1B). Participants were more often women (56%–57%); of native Dutch origin (88%–89%), as compared to Western (7%–9%) and non‐Western (3%–4%); and of lower urbanization degree (49%–50%), versus moderate (31%–32%) or high (19%–20%). Educational level (high vs. middle/low) was nearly equally divided each round. One third could be classified as having comorbidities in 2021, which slightly increased to 4 out of 10 in 2022. The majority of LVC‐participants were of native Dutch, low urbanization degree (both > 97%), low/middle educational level (71%–73%), and median age as well as the proportion of having comorbidities (18%–24%) was lower than in NL.

**TABLE 1:** Description of the PIENTER Corona (PICO) study cohort in 2021 and 2022, covering study rounds 4 to 9.

		Feb 2021 (PICO4)		June 2021 (PICO5)		Nov 2021 (PICO6)		Mar 2022 (PICO7)		June 2022 (PICO8)		Nov 2022 (PICO9)	
		*n*	*%*	*n*	*%*	*n*	*%*	*n*	*%*	*n*	*%*	*n*	*%*
**National sample**													
All ages		5981	100.0	5335	100.0	8138	100.0	6527	100.0	5823	100.0	5371	100.0
Working age (18–67 year)		4088	68.3	3614	67.7	4811	59.1	3775	58.3	3299	56.7	3046	56.7
Sex	Men	2630	44.0	2322	43.5	3527	43.3	2844	43.6	2546	43.7	2339	43.5
	Women	3351	56.0	3013	56.5	4611	56.7	3683	56.4	3277	56.3	3032	56.5
Age (median years [IQR])		51 (31–65)		53 (34–66)		51 (29–69)		54 (33–70)		56 (36–71)		57 (38–72)	
Ethnic background	Dutch	5290	88.4	4727	88.6	7182	88.3	5799	88.9	5150	88.4	4760	88.6
	Western	453	7.6	397	7.4	669	8.2	523	8.0	492	8.5	443	8.3
	Non‐Western	238	4.0	211	4.0	287	3.5	205	3.1	181	3.1	168	3.1
Educational level (≥ 25 years)	High	2365	47.9	2149	47.2	3148	48.7	2598	48.4	2343	47.7	2180	48.4
	Middle/Low	2574	52.1	2379	52.5	3320	51.3	2766	51.6	2564	52.3	2468	51.6
Region	North	1183	19.8	1047	19.6	1642	20.2	1337	20.5	1183	20.3	1077	20.1
	Mid‐West	1063	17.8	952	17.8	1475	18.1	1159	17.8	1068	18.3	991	18.4
	Mid‐East	1277	21.3	1118	21.0	1722	21.2	1397	21.4	1220	21.0	1118	20.8
	South‐West	1094	18.3	984	18.4	1502	18.5	1199	18.4	1078	18.5	987	18.4
	South‐East	1364	22.8	1234	23.1	1797	22.1	1435	22.0	1274	21.9	1198	22.3
Urbanization degree	High (large cities)	1144	19.1	1026	19.2	1598	19.6	1263	19.3	1114	19.1	1022	19.0
	Middle (moderate cities)	1892	31.6	1674	31.4	2550	31.3	2.029	31.1	1814	31.2	1681	31.3
	Low (villages to countryside)	2945	49.2	2635	49.4	3990	49.0	3.235	49.6	2895	49.7	2668	49.7
Comorbidities	Yes	1872	32.0	1730	33.8	2731	34.7	2730	42.1	2550	44.2	2393	44.9
	No	3970	68.0	3390	66.2	5131	65.3	3750	57.9	3215	55.8	2931	55.1
COVID‐19 vaccination (among ≥ 18 years)	Yes (≥ 1 dose)	266	5.2	3945	83.9	6602	96.5	5418	97.4	4876	97.5	4627	97.4
	No	4870	94.8	755	16.1	240	3.5	147	2.6	126	2.5	123	2.6
No. of COVID‐19 vaccine doses	1	142	2.8	1645	35.0	651	9.5	83	1.5	59	1.2	57	1.2
	2	124	2.4	2298	48.9	5850	85.6	786	14.1	596	11.9	467	9.8
	3	NA	NA	NA	NA	93	1.4	3944	70.9	2790	55.8	1428	30.1
	4	NA	NA	NA	NA	NA	NA	604	10.8	1403	28.0	1297	27.3
	5	NA	NA	NA	NA	NA	NA	1	< 0.1	29	0.6	1346	28.3
	6	NA	NA	NA	NA	NA	NA	NA	NA	NA	NA	30	0.6
**LVC sample**													
All ages		470	100.0	432	100.0	411	100.0	322	100.0	303	100.0	275	100.0
Sex	Men	187	39.8	169	39.2	167	40.6	137	42.5	127	41.9	118	42.9
	Women	283	60.2	263	60.9	244	59.4	185	57.5	176	58.1	157	57.1
Age (median years [IQR])		44 (28–58)		46 (30–59)		47 (32–60)		50 (36–63)		51 (37–64)		52 (38–65)	
COVID‐19 vaccination (among ≥ 18 years)	Yes (≥ 1 dose)	19	4.8	249	67.3	292	81.3	238	82.9	224	83.0	206	83.4
	No	379	95.2	121	32.7	67	18.7	49	17.1	46	17.0	41	16.6
No. of COVID‐19 vaccine doses	1	10	2.5	124	33.5	43	12.0	8	2.8	7	2.6	7	2.8
	2	9	2.3	125	33.8	248	69.1	60	20.9	53	19.6	44	17.8
	3	NA	NA	NA	NA	1	0.3	154	53.7	125	46.3	80	32.4
	4	NA	NA	NA	NA	NA	NA	16	5.6	39	14.5	38	15.4
	5	NA	NA	NA	NA	NA	NA	NA	NA	NA	NA	37	15.0
	6	NA	NA	NA	NA	NA	NA	NA	NA	NA	NA	NA	NA

*Note:* Numbers (*n*) and proportion (%) were provided for participants in the current PIENTER Corona (PICO) study cohort belonging to the national sample and low vaccination coverage (LVC) sample, covering study rounds in 2021 (February [PICO4], June [PICO5] and November [PICO6]) and 2022 (March [PICO7], June [PICO8], November [PICO9]), by sociodemographic characteristics, and comorbidity‐ and vaccination status. Educational level (from 25 years of age, highest obtained or current) was classified as low (no education or primary education)/middle (secondary school or vocational training), or high (bachelor's degree, university). Regions consist of the following provinces: North = Groningen, Friesland, Drenthe and Overijssel; Mid‐West = Flevoland and Noord‐Holland; Mid‐East = Gelderland and Utrecht; South‐West = Zuid‐Holland and Zeeland; South‐East = Noord‐Brabant and Limburg. Those who had received a flu vaccination were classified as having underlying comorbidities, consistent with national regulation. NA = not applicable.

COVID‐19 vaccination coverage as well as the number of doses followed a distinct age‐pattern over time in NL, consistent with the vaccine roll‐out (Figure S2). Overall weighted vaccination coverage (≥ 1 dose) in ≥ 18 years was 5% (95% CI 4–6) in February 2021, rose steeply to 78% (95% CI 76–80) in June, and increased further to 96% (95% CI 95–97) in November 2021. In March 2022, 78% (95% CI 77–80) had received a booster dose, and in June 18% (95% CI 17–20) had been administered a second booster. The latter increased to 43% (95% CI 41–44) in November, while 17% (95% CI 16–19) had received a fifth dose. Overall coverage did not differ much between sexes, yet was slightly higher in women < 60 years in June 2021 for the primary series, but this difference was nearly erased by the end of the year and coverage remained stable for the doses thereafter. Uptake was consistently lower in the LVC (Table 1).

### 3.2 Infection‐Induced and Total Seroprevalence

#### 3.2.1 Overall, by Age and Region

**FIGURE 2:**
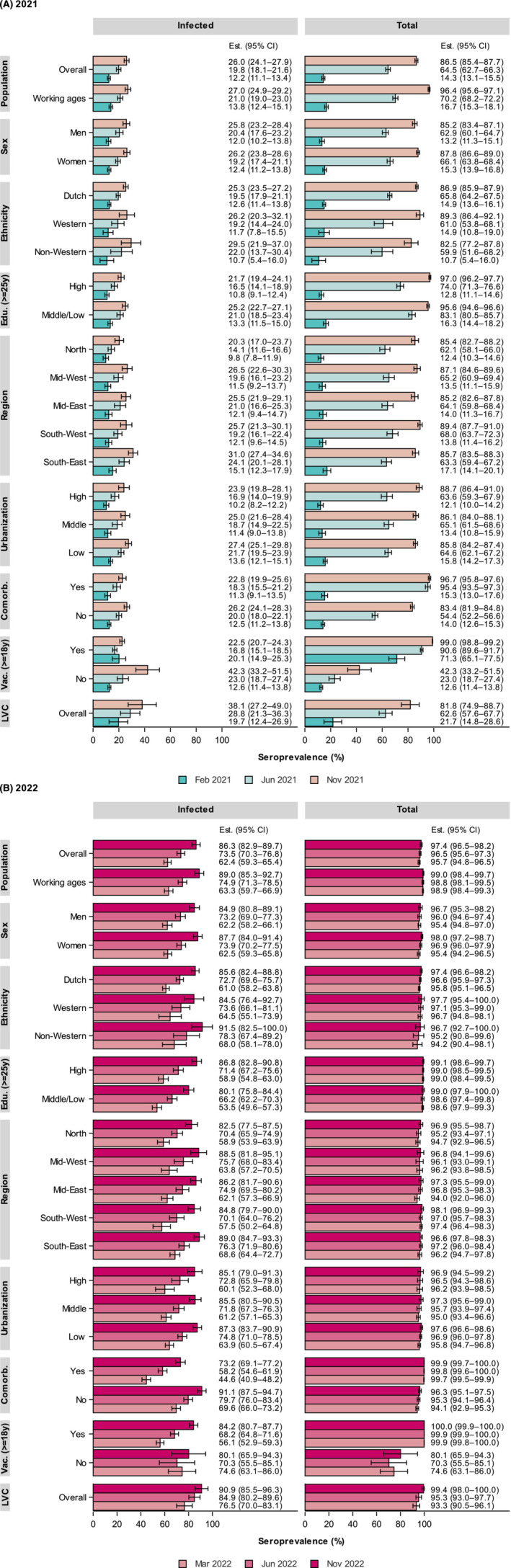
Weighted SARS‐CoV‐2 seroprevalence (with 95% confidence intervals [CI]) induced by infection (left panel) and total (i.e., infection and vaccination, right panel) in the general Dutch population in 2021 (February (PICO4), June (PICO5) and November (PICO6)) (A) and 2022 (March [PICO7], June [PICO8], November [PICO9]) (B), by sociodemographic characteristics, comorbidity‐, and vaccination (≥ 18 years) status. Regions consist of the following provinces: North = Groningen, Friesland, Drenthe and Overijssel; Mid‐West = Flevoland and Noord‐Holland; Mid‐East = Gelderland and Utrecht; South‐West = Zuid‐Holland and Zeeland; South‐East = Noord‐Brabant and Limburg. Comorb. = having comorbidities, that is, classification based on having received the annual flu vaccination, consistent with national regulation; Edu. = educational level (from 25 years of age, highest obtained or current), classified as low (no education or primary education)/middle (secondary school or vocational training), or high (bachelor's degree, university); Est. (95% CI) = seroprevalence estimate with 95% confidence intervals; LVC = low vaccination coverage sample, as conceived in the PIENTER‐3 cohort); Population = general Dutch population, stratified by all ages (overall) and working ages (18–67 years); Vac. = had a COVID‐19 vaccination at least once (from 18 years of age).

**FIGURE 3:**
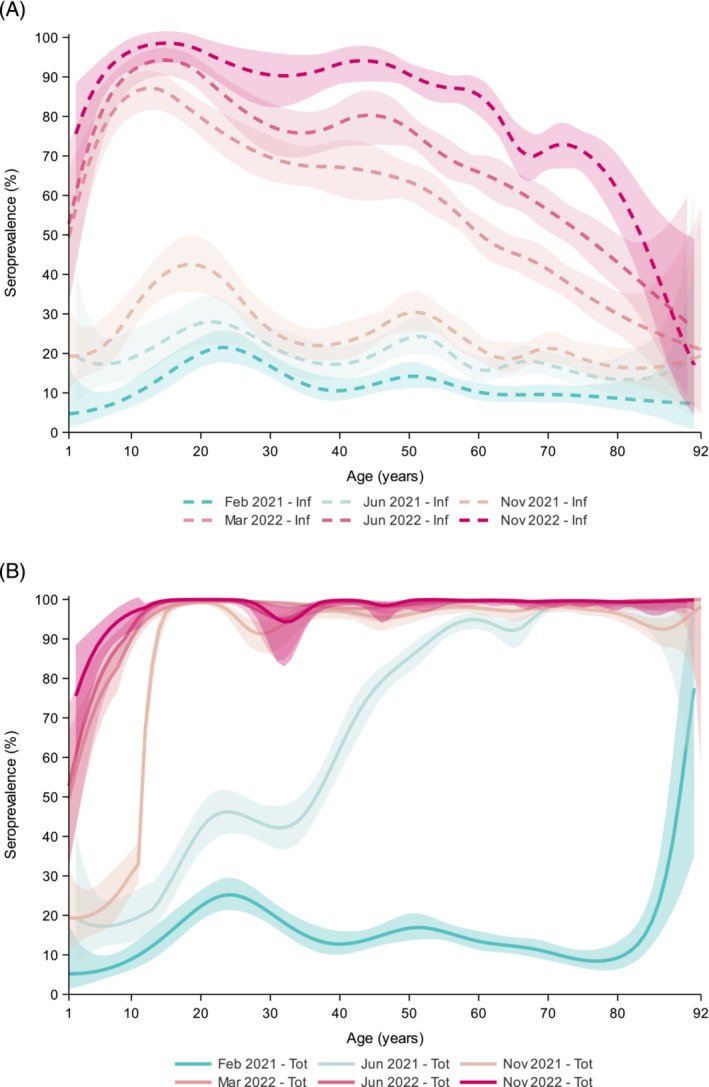
Weighted SARS‐CoV‐2 seroprevalence (with 95% confidence bands) induced by infection (A) and total (i.e., infection and vaccination) (B) in the general Dutch population in 2021 (February [PICO4], June [PICO5] and November [PICO6]) and 2022 (March [PICO7], June [PICO8], November [PICO9]), by age. Seroprevalence was modeled with B splines, with number of percentile‐placed knots optimized following Akaike's information criterion (AIC).

The overall and stratified, inf‐ and total seroprevalence are displayed in Figure 2 for 2021 (A) and 2022 (B), and by age in Figure 3 (A: inf; B: total).

In February 2021, pre‐Alpha, the total seroprevalence reached 14% (95% CI 13–16) in the Dutch population and already spiked in the oldest ages, predominantly due to the primary series vaccination. This continued with rapid pace during Alpha, rising overall to 65% (95% CI 63–66) in June, and among ≥ 50‐years‐olds to > 80%. Meanwhile, overall inf‐seroprevalence rose from 12% (95% CI 11–13) to 20% (95% CI 18–22), and displayed a sustained age‐pattern with highest estimates in young adults (30%), followed by middle‐aged adults (25%), and lowest in elderly (15%). Amidst Delta in November, total seroprevalence was high (87% (95% CI 85–88)) and reached > 90% in ≥ 12‐year‐olds. Inf‐seroprevalence increased further to 26% (95% CI 24–28), with a relative steep rise in the age range from primary school to young adults, peaking around 40%, consistent with a relative low vaccination coverage.

In 2022, total seroprevalence already reached 96% (95% CI 95–97) at the beginning of the year and remained high thereafter. Inf‐seroprevalence increased sharply to 62% (95% CI 59–65) in March after emergence of Omicron‐BA.1. Rapid expansion was especially noticeable in < 60‐year‐olds, with estimates around 50% in the youngest ages and up to 90% in adolescents, from where it decreased linearly with age (e.g., 50% in 60‐year‐olds and 30% in 80‐year‐olds). After easing of most control measures in spring, overall inf‐seroprevalence rose further to 74% (95% CI 70–77) in June following Omicron‐BA.2. While nearly reaching 100% in adolescents, relative steepest increases were now seen in > 40‐year‐olds (up to 80% in 50‐year‐olds and 40% in 80‐year‐olds), despite high booster vaccination uptake earlier. In November, following Omicron‐BA.5, inf‐seroprevalence increased further to 86% (95% CI 83–90) in NL, and > 90% among those aged < 60 years. Regardless of this relative large increase in adults during the second half of 2022, still 30% of those vaccinated aged ≥ 60 years lacked hybrid immunity, and 50% ≥ 80 years.

Most infections pre‐Alpha were observed in the South‐East region (15%) and fewest in the North (10%) (Figure S3 displays all 25 Municipality Health Service [GGD] regions). A fairly similar picture was extended across 2021, with steepest increase in the South‐East (31%) and Mid‐West (27%), and lowest in the North (20%, *p* < 0.001 and *p* = 0.04, respectively). Low urbanized areas had the highest observed inf‐seroprevalence throughout the study period, but most pronounced in June 2021 following Alpha (22% vs. high: 17%; *p* = 0.007). Generally, regional differences did not further intensify in 2022. In LVC, inf‐seroprevalence was considerably higher than in NL, especially noticeable till the end of 2021 (~1.5 times: 38% vs. 26%, *p* = 0.01), and reached > 90% after Omicron‐BA.5 at the end of 2022. Correspondingly, the total seroprevalence was nearly consistently lower in that period, illustrative of the lower COVID‐19 vaccination coverage.

#### 3.2.2 (Sub)Groups

Both inf‐ and total seroprevalence did not differ much between sexes. Interestingly, middle‐aged women (50–59 years) were significantly more often infected than men already pre‐Alpha (16% vs. 9%, respectively, *p* < 0.001) and remained higher throughout 2021–2022 (Figure S4). Conversely, men aged ≥ 75 years had on average two times higher inf‐seroprevalence up until the beginning of 2022 (39% vs. 24% in women, *p* < 0.001).

Among persons of non‐Western descent, inf‐seroprevalence rose ~1.5 times sharper during Alpha than in Western and native Dutch, and this was particularly noticeable among < 40‐year‐olds (Figure S5). Higher inf‐seroprevalence persisted among non‐Western in all subsequent waves, yet the total seroprevalence was consistently lowest in this group. Moreover, low/middle‐educated persons (from age 25 years) had a significantly higher inf‐seroprevalence compared to high‐educated already pre‐Alpha and throughout 2021 (e.g., June: 21% vs. 17%, *p* = 0.006). This was consistent for all age groups (Figure S6), but most noticeable in individuals aged ≥ 60 years (Alpha: 18% vs. 12%, respectively, *p* < 0.001; and amidst Delta: 22% vs. 13%, respectively, *p* = 0.002), coinciding with a lower total seroprevalence in June (*p* = 0.03). Age group‐specific seroprevalence estimates became more comparable after the emergence of Omicron. Further, in persons with underlying comorbidities, targeted for early COVID‐19 vaccination, a very high total seroprevalence (> 95%) was already observed in June 2021. Inf‐seroprevalence continued to be relatively low in this group compared to those without up until March 2022 after Omicron BA.1 (45% vs. 70%, *p* < 0.001), yet increased with higher rate thereafter (e.g., after Omicron‐BA.5 in November 2022: 73% vs. 91%).

#### 3.2.3 Occupations

**FIGURE 4:**
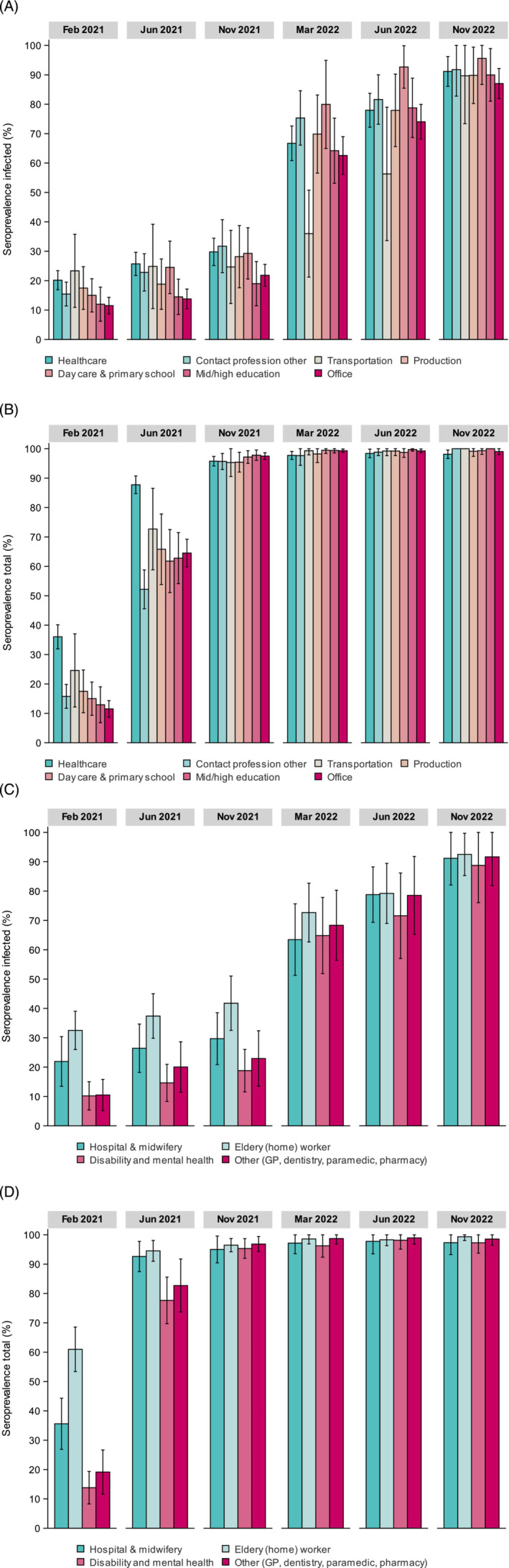
Weighted SARS‐CoV‐2 seroprevalence (with 95% confidence intervals) induced by infection (A, C) and total (i.e., infection and vaccination) (B, D) in the general Dutch population in 2021 (February (PICO4), June (PICO5) and November (PICO6)) and 2022 (March (PICO7), June (PICO8), November (PICO9)), by age categories (years) and occupational sector (A, B) and healthcare sector (C, D). Contact professions consisted of sectors outside healthcare, such as bars, hotels, restaurants, stores (retail and supermarket), barbers, tattooing, masseuse, cosmetologist, etc. The median age in years (IQR) per occupational sector (in PICO6, as reference) was healthcare: 48 (35–57); contact profession: 43 (22–55); transportation: 55 (37–60); production: 49 (37–58); day care & primary school: 45 (35–56); middle & higher education: 49 (33–60); and office: 47 (36–55).

Inf‐seroprevalence in the working‐age population (18–67 years) followed a similar pattern as the general population throughout 2021–2022. HCW had among the highest inf‐seroprevalence pre‐Alpha (20%)—together with transportation and production sectors (Figure 4A,B)—which was mainly due to high rates in elderly (home)workers (33%) and hospital employees (22%) (Figure 4C,D). Congruent with a very high total seroprevalence already in June 2021 (> 90%) due to prioritization of vaccination, the rate of infections in HCW slowed down (after Alpha: 26%; amidst Delta: 30%) relative to nationwide, and was similar in March 2022 after Omicron‐BA.1. Inf‐seroprevalence rates in other HCW sectors were relatively moderate to low until emergence of Omicron‐BA.1. Furthermore, sharpest inf‐seroprevalence elevations in 2021 were seen among those working in contact professions other than HCW (from 15% in February to 32% in November) and day‐care & primary education (from 15% to 29%), and these trends extended into the Omicron waves (e.g., after Omicron‐BA.2 [June 2022]: 82% and 91%, respectively; to note, the median age was lowest in these sectors: between 43 and 45 years, vs. others sectors 47–55). Conversely, office employees and those working in middle/higher education had nearly two times lower estimates than HCW after Alpha in June 2021, and only started to catch‐up with countrywide estimates thereafter, particularly since emergence of Omicron.

### 3.3 Breakthrough Infections

**FIGURE 5:**
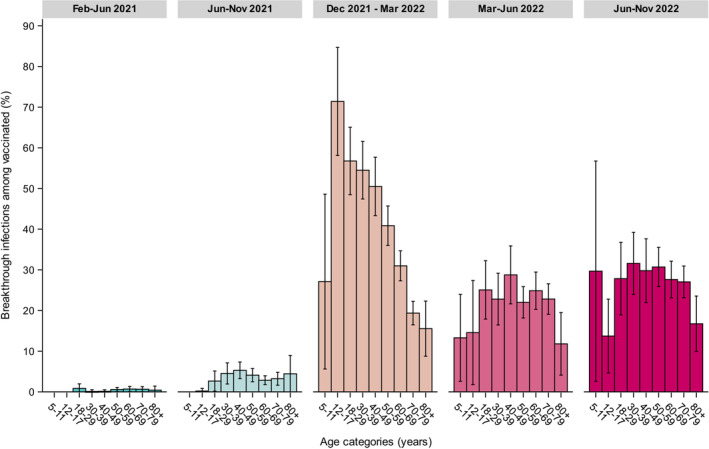
Weighted SARS‐CoV‐2 breakthrough infections (with 95% confidence intervals) in the general vaccinated Dutch population in 2021 (between February [PICO4], June [PICO5] and November [PICO6]) and 2022 (between March [PICO7], June [PICO8], November [PICO9]), by age categories (years).

Primarily in 2021, inf‐seroprevalence was significantly lower among adult vaccinees, for example, amidst Delta in November 2021 (22% vs. 42% among unvaccinated, *p* < 0.001) (Figure 2) (to note, in February 2021, the higher inf‐seroprevalence among vaccinated is due to the high proportion of HCW who had been infected relative more often than the average population pre‐Alpha, *p* = 0.001). This can be explained by presence of only few breakthrough infections detected (February–June 2021, Alpha: 0.5% [95% CI 0.2–0.7]; and June–November 2021, amidst Delta: 3.5% [95% CI 2.9–4.3]). Inf‐seroprevalence elevated with rather similar rate among vaccinated and unvaccinated since emergence of Omicron‐BA.1, and even steeper in vaccinated after Omicron‐BA.2 (mid‐2022, from 56% to 68%). These data correspond to the large proportion of breakthrough infections observed among vaccinated since Omicron (December 2021–March 2022 (Omicron‐BA.1): 43% [95% CI 40–46]; March–June 2022 (Omicron‐BA.2): 23% [95% CI 21–26]; June–November 2022 (Omicron‐BA.5): 28% [95% CI 25–30]), at first mainly among adolescents up until middle‐aged adults, but since Omicron‐BA.2 equally across adults up to 80 years (Figure 5).

## 4 Discussion

This large Dutch nationwide population‐based prospective seroepidemiological study supported public health decision‐making during the COVID‐19 pandemic, for example, as input for the Outbreak Management Team, Ministry of Health, Dutch Health Council, and forecasting/modeling purposes. Our study underscores the importance of serosurveillance globally to supplement other tools in better understanding population immunity and susceptibility over time. The total seroprevalence in NL increased rapidly during the VOC‐era, especially in persons prioritized for vaccination. However, infection rates were unequally distributed between (sub)groups and regions pre‐Omicron, with highest rates noticeable in adolescents and young adults, low/middle educated elderly, non‐Western, those in contact professions including working in childcare, the South‐East region and LVC. Following multiple waves of Omicron, the number of breakthrough infections increased steeply, resulting in hybrid immunity for the majority of the Dutch population by the end of 2022. Nevertheless, approximately 3 out of 10 vaccinated vulnerable individuals (i.e., elderly and those with comorbidities) had not acquired hybrid immunity, necessitating tailored vaccination efforts to reduce the risk of severe disease upon infection and ongoing seromonitoring.

Our population‐based results are indicative of the prevention of infections by vaccination pre‐Omicron and in line with vaccine‐effectiveness studies as well as seroepidemiological studies conducted in regions with high vaccination coverage [12]. Particular illustrative was the relative modest increase in infections in HCW since vaccination despite having the highest exposure—potentially in combination with adequate use of personal protective equipment. Conversely, the rate of infection remained high in the South‐Eastern part of the country, that is, the epicenter of the first wave in 2020, and particularly in LVC [2]. A combination of lower vaccine uptake (consistent with lower total seroprevalence) and increased exposure, for example, due to lower adherence to measures, might have caused this and has also been observed in similar communities abroad, such as the United Kingdom [4, 22]. Future research should gain more understanding of the concerns in harder‐to‐reach regions and apply this knowledge as input for tailor‐made preventive programs. Further, in‐depth research on air quality across regions and severity of COVID‐19 has shown an increased risk in the lower urbanized areas in the South‐East, recognized for poorer air quality due to intensive livestock farming [23].

Importantly, relatively higher rates of infection were also observed in low/middle educated elderly and non‐Western, particularly after roll‐out of vaccination. The pandemic entails a double burden for the most disadvantaged groups: increased likelihood of infection, for example, due to fewer opportunities to work remotely, as well as severe disease due to weaker health or access to health. Our data are congruent with lower vaccination uptake and higher ICU admissions in these specific groups in our country and abroad during this period, such as other high‐income countries as Switzerland and the United States [4, 24, 25]. Global efforts are needed to tackle mis‐information and apply tailor‐made approaches for vaccination, especially since coverage against vaccine‐preventable diseases in general is declining.

A distinct age‐pattern was seen up until 2021 with infections peaking in adolescents, young adults and middle‐aged women. While nationwide stringent control measures were still in effect, primary schools remained open during emergence of VOCs. As opposed to what we observed pre‐Alpha [3], the rate of infections in the youngest age groups rose quickly which corresponds to reports on increased transmissibility and susceptibility in children since emergence of VOCs [26], with spikes in incidence in these age groups observed in countries worldwide [5, 16, 27]. Consistent herewith, inf‐seroprevalence elevated in adults working in childcare/primary schools. Pre‐Alpha, highest rates of infections were seen in those working in transportation, production and healthcare. Steepest increases were later observed in those working in other contact professions, such as physiotherapists, barbers and store employees, whereas office employees were still among the lowest infected, presumably due to working remotely. Also, despite the highest rates in young adults, professions in middle/higher education were comparable to nationwide estimates. However, unless demonstrative beneficial effects of limited contact and remote schooling on reducing viral transmission, potential detrimental social‐ and cognitive effects should be taken into account while evaluating the pandemic response [28].

To our knowledge this is the first study to present population estimates of breakthrough infections across various VOCs. After the first Omicron wave and relaxation of control measures, the rate of infections had increased steeply across the population, consistent with reports globally [16], and differences in seroprevalence between groups shrunk. A large elevation in the proportion of breakthrough infections was observed, firstly among adolescents up until middle‐aged adults and in later waves across all adults. This trend is most likely explained by increased booster doses in elderly, which have shown to be effective against infection in the short term, but susceptibility increased due to waning immunity alongside novel variants with enhanced immune‐escape features [12]. Hybrid immunity has proven to be most effective against future infection [29], potentially mediated by mucosal antibodies in the upper respiratory tract [30, 31], and severe disease [32]. These observations on susceptibility in the most vulnerable persons contributed to targeted prevention strategies with novel booster doses for winter 2023, and warrants ongoing seromonitoring (of vaccine‐effectiveness).

This study has several strengths and limitations that should be highlighted. This is one of the largest prospective population‐based serological cohorts, including children and a unique LVC sample, that was set‐up since the beginning of the pandemic and is still ongoing. Since (self‐)testing has been reduced significantly after relaxation of control measures in 2022, serological assessment including boosting of antibodies, is essential for detection of (breakthrough) infections, and for guiding surveillance. Anti‐N generally has a somewhat lower sensitivity than anti‐S1 and is known to wane quicker [19, 33] which might have caused some underestimation of breakthrough infections. However, we did not observe a significant reduction in sensitivity of anti‐N in vaccinees fortunately [34], which has been reported by some [35], and due to our repeated sampling with short intervals we still expect high case ascertainment. Moreover, in‐depth questionnaire data allowed investigation into (sub)groups. Response rates in children were rather low and drop‐out rates high, however since children were less restricted by control measures and schools were mostly open, we do not expect large differences between children in terms of exposure, and this was also confirmed by contact data [36, 37]. Despite random selection and weighting our sample, some groups are underrepresented, such as those living in nursing homes—that were hit hard pre‐Alpha—and non‐Western, who might have refrained from participation due to digital‐ and/or language barriers. Study participants may generally adhere better to control measures, also reflected by a higher vaccination coverage than the general population, which could have underestimated inf‐seroprevalence especially pre‐Omicron.

To conclude, this seroepidemiological study has provided important insights on dynamics of SARS‐CoV‐2 population immunity resulting from (breakthrough) infections and vaccination during the VOC‐era. These results will be key in evaluation of control measures, construction of future pandemic response planning, and guiding ongoing preventive strategies for the most vulnerable groups. In the endemic/post‐pandemic phase, this robust serological framework will be vital for the relevant challenges ahead, such as unraveling risk factors for post‐COVID and mucosal immunity, but also in gaining knowledge on the (potentially permanent) epidemiological changes observed for other respiratory pathogens during the pandemic, for example, respiratory syncytial virus and group A streptococcal [38, 39].

## Author Contributions

**Eric R.A. Vos:** conceptualization, investigation, writing–original draft; methodology, visualization, software, formal analysis. **Cheyenne C.E. van Hagen:** validation, visualization, writing–review and editing. **Denise Wong:** project administration, data curation, resources. **Gaby Smits:** validation, data curation, resources. **Marjan Kuijer:** validation, data curation, resources. **Alienke J. Wijmenga‐Monsuur:** data curation, project administration, writing–review and editing, resources. **Joanna Kaczorowska:** writing–review and editing. **Robert S. van Binnendijk:** conceptualization, writing–review and editing. **Fiona R.M. van der Klis:** conceptualization; funding acquisition, writing–review and editing. **Gerco den Hartog:** conceptualization, writing–review and editing. **Hester E. de Melker:** conceptualization, funding acquisition, supervision, writing–review and editing.

## Ethics Statement

The study was approved by the Medical Ethics Committee MEC‐U, the Netherlands (Clinical Trial Registration NTR8473), conformed to the principles embodied in the Declaration of Helsinki, and all participants (or parents/guardians) provided written informed consent.

## Conflicts of Interest

The authors declare no conflicts of interest.

### Peer Review

The peer review history for this article is available at https://www.webofscience.com/api/gateway/wos/peer‐review/10.1111/irv.13312.

## Supporting information


**Table S1** Overview of responders vs. non‐responders from the additional sampling in Nov. 2021 (PICO6).
**Figure S1.** Number of participants in the current PIENTER Corona (PICO) study belonging to the national sample and low vaccination coverage (LVC) sample (as conceived in the PIENTER‐3 cohort) in 2021 (February (PICO4), June (PICO5) and November (PICO6)) and 2022 (March (PICO7), June (PICO8), November (PICO9)), by age categories (years) and region. Regions consist of the following provinces:North = Groningen, Friesland, Drenthe and Overijssel; Mid‐West = Flevoland and Noord‐Holland; Mid‐East = Gelderland and Utrecht; South‐West = Zuid‐Holland and Zeeland; and South‐East = Noord‐Brabant and Limburg.
**Figure S2.** Weighted vaccination coverage (%) in the general Dutch population (as derived from the national sample of the current PIENTER Corona (PICO) study cohort) in 2021 (February (PICO4), June (PICO5) and November (PICO6)) and 2022 (March (PICO7), June (PICO8), November (PICO9)), by sex, age categories (years) and number of vaccine doses. The vaccination campaign roll‐out followed an age‐, comorbidity‐, and healthcare worker‐based prioritization that started in the beginning of 2021.
**Figure S3.** Weighted SARS‐CoV‐2 seroprevalence induced by infection (**a**) and total (i.e., infection and vaccination) (**b**) in the general Dutch population in 2021 (February (PICO4), June (PICO5) and November (PICO6)) and 2022 (March (PICO7), June (PICO8), November (PICO9)), by Municipality Health Service (GGD) region.
**Figure S4.** Weighted SARS‐CoV‐2 seroprevalence (with 95% confidence intervals) induced by infection (**a**) and total (i.e., infection and vaccination) (**b**) in the general Dutch population in 2021 (February (PICO4), June (PICO5) and November (PICO6)) and 2022 (March (PICO7), June (PICO8), November (PICO9)), by age categories (years) and sex.
**Figure S5.** Weighted SARS‐CoV‐2 seroprevalence (with 95% confidence intervals) induced by infection (**a**) and total (i.e., infection and vaccination) (**b**) in the general Dutch population in 2021 (February (PICO4), June (PICO5) and November (PICO6)) and 2022 (March (PICO7), June (PICO8), November (PICO9)), by age categories (years) and ethnic background.
**Figure S6.** Weighted SARS‐CoV‐2 seroprevalence (with 95% confidence intervals) induced by infection (**a**) and total (i.e., infection and vaccination) (**b**) in the general Dutch population in 2021 (February (PICO4), June (PICO5) and November (PICO6)) and 2022 (March (PICO7), June (PICO8), November (PICO9)), by age categories (years) and educational level ((from 25 years of age, highest obtained or current). Educational level was classified as low (no education or primary education)/middle (secondary school or vocational training), or high (bachelor’s degree, university)).

## Data Availability

Upon reasonable request to the principal investigator.

## References

[1] Statistics Netherlands (CBS) , “Statline: Cause of Death 2021,” Available from: https://opendata.cbs.nl/#/CBS/en/dataset/7233ENG/table.

[2] E. R. A. Vos , G. den Hartog , R. M. Schepp , et al., “Nationwide Seroprevalence of SARS‐CoV‐2 and Identification of Risk Factors in the General Population of the Netherlands During the First Epidemic Wave,” Journal of Epidemiology and Community Health 75 (2020): 489–495. 33249407 10.1136/jech-2020-215678 PMC8142429

[3] E. R. A. Vos , M. van Boven , G. den Hartog , et al., “Associations Between Measures of Social Distancing and SARS‐CoV‐2 Seropositivity: A Nationwide Population‐Based Study in the Netherlands,” Clinical Infectious Diseases 73 (2021): 2318–2321. 33772265 10.1093/cid/ciab264 PMC8083720

[4] Valk A , van Meijeren D , Smorenburg N , et al., “COVID‐19 Vaccination Coverage in the Netherlands in 2021,” National Institute for Public Health and the Environment (RIVM), (2022).

[5] F. Campbell , B. Archer , H. Laurenson‐Schafer , et al., “Increased Transmissibility and Global Spread of SARS‐CoV‐2 Variants of Concern as at June 2021,” Eurosurveillance 26, no. 24 (2021): 2100509. 34142653 10.2807/1560-7917.ES.2021.26.24.2100509 PMC8212592

[6] P. Bager , J. Wohlfahrt , J. Fonager , et al., “Risk of Hospitalisation Associated With Infection With SARS‐CoV‐2 Lineage B.1.1.7 in Denmark: An Observational Cohort Study” The Lancet Infectious Diseases 21 (2021): 1507–1517. 34171231 10.1016/S1473-3099(21)00290-5 PMC8219488

[7] K. A. Twohig , T. Nyberg , A. Zaidi , et al., “Hospital Admission and Emergency Care Attendance Risk for SARS‐CoV‐2 Delta (B.1.617.2) Compared With Alpha (B.1.1.7) Variants of Concern: A Cohort Study,” The Lancet Infectious Diseases 22 (2022): 35–42. 34461056 10.1016/S1473-3099(21)00475-8 PMC8397301

[8] P. T. de Boer , J. van de Kassteele , E. R. A. Vos , et al., “Age‐Specific Severity of Severe Acute Respiratory Syndrome Coronavirus 2 in February 2020 to June 2021 in the Netherlands” Influenza and Other Respiratory Viruses 17, no. 8 (2023): e13174. 37621921 10.1111/irv.13174 PMC10444602

[9] B. de Gier , L. van Asten , T. M. Boere , et al., “Effect of COVID‐19 Vaccination on Mortality by COVID‐19 and on Mortality by Other Causes, the Netherlands, January 2021‐January 2022,” Vaccine 41, no. 31 (2023): 4488–4496. 37328352 10.1016/j.vaccine.2023.06.005 PMC10247887

[10] Dutch Government , “Corona Dashboard 2023,” Available from: https://coronadashboard.rijksoverheid.nl/.

[11] J. Pijpers , A. van Roon , C. van Roekel , et al., “Determinants of COVID‐19 Vaccine Uptake in the Netherlands: A Nationwide Registry‐Based Study,” Vaccine 11, no. 9 (2023): 1409. 10.3390/vaccines11091409 PMC10537724 37766087

[12] D. R. Feikin , M. M. Higdon , L. J. Abu‐Raddad , et al., “Duration of Effectiveness of Vaccines Against SARS‐CoV‐2 Infection and COVID‐19 Disease: Results of a Systematic Review and Meta‐Regression,” The Lancet 399, no. 10328 (2022): 924–944. 10.1016/S0140-6736(22)00152-0 PMC8863502 35202601

[13] A. J. Huiberts , B. de Gier , C. E. Hoeve , et al., “Vaccine Effectiveness of Primary and Booster COVID‐19 Vaccinations Against SARS‐CoV‐2 Infection in the Netherlands From 12 July 2021 to 6 June 2022: A Prospective Cohort Study,” International Journal of Infectious Diseases 133 (2023): 36–42. 37086863 10.1016/j.ijid.2023.04.401 PMC10118053

[14] National Institute for Public Health and the Environment (RIVM) , “Vaccination Figures for Autumn 2022 Round of Covid‐19 Repeat Vaccination Bilthoven: RIVM; 2023 [updated August 23, 2023,” Available from: https://www.rivm.nl/en/covid‐19‐vaccination/figures‐vaccination‐programme.

[15] S. A. McDonald , F. Miura , E. R. A. Vos , et al., “Estimating the Asymptomatic Proportion of SARS‐CoV‐2 Infection in the General Population: Analysis of Nationwide Serosurvey Data in the Netherlands,” European Journal of Epidemiology 36 (2021): 735–739. 34114187 10.1007/s10654-021-00768-y PMC8191704

[16] I. Bergeri , M. G. Whelan , H. Ware , et al., “Global SARS‐CoV‐2 Seroprevalence From January 2020 to April 2022: A Systematic Review and Meta‐Analysis of Standardized Population‐Based Studies,” PLoS Medicine 19, no. 11 (2022): e1004107. 36355774 10.1371/journal.pmed.1004107 PMC9648705

[17] J. D. M. Verberk , R. A. Vos , L. Mollema , et al., “Third National Biobank for Population‐Based Seroprevalence Studies in the Netherlands, Including the Caribbean Netherlands,” BMC Infectious Diseases 19, no. 1 (2019): 470. 31138148 10.1186/s12879-019-4019-y PMC6537387

[18] G. den Hartog , R. M. Schepp , M. Kuijer , et al., “SARS‐CoV‐2–Specific Antibody Detection for Seroepidemiology: A Multiplex Analysis Approach Accounting for Accurate Seroprevalence,” The Journal of Infectious Diseases 222, no. 9 (2020): 1452–1461. 32766833 10.1093/infdis/jiaa479 PMC7454740

[19] L. L. van den Hoogen , G. Smits , C. C. E. van Hagen , et al., “Seropositivity to Nucleoprotein to Detect Mild and Asymptomatic SARS‐CoV‐2 Infections: A Complementary Tool to Detect Breakthrough Infections After COVID‐19 Vaccination?” Vaccine 40, no. 15 (2022): 2251–2257. 35287986 10.1016/j.vaccine.2022.03.009 PMC8904156

[20] W. J. Rogan and B. Gladen , “Estimating Prevalence From the Results of a Screening Test,” American Journal of Epidemiology 107, no. 1 (1978): 71–76. 623091 10.1093/oxfordjournals.aje.a112510

[21] P. J. Bickel and K. A. Doksum , Mathematical Statistics: Basic Ideas and Selected Topics, Volumes I‐II Package (New York: CRC Press, 2015).

[22] K. M. Gaskell , M. Johnson , V. Gould , et al., “SARS‐CoV‐2 Seroprevalence in a Strictly‐Orthodox Jewish Community in the UK: A Retrospective Cohort Study,” The Lancet Regional Health ‐ Europe 6 (2021): 100127. 34308409 10.1016/j.lanepe.2021.100127 PMC8291041

[23] J. Zorn , M. Simões , G. J. M. Velders , et al., “Effects of Long‐Term Exposure to Outdoor Air Pollution on COVID‐19 Incidence: A Population‐Based Cohort Study Accounting for SARS‐CoV‐2 Exposure Levels in the Netherlands,” Environmental Research 252 (2024): 118812. 38561121 10.1016/j.envres.2024.118812

[24] Racial and Ethnic Disparities in COVID‐19–Related Infections, Hospitalizations, and Deaths,” Annals of Internal Medicine 174, no. 3 (2021): 362–373. 33253040 10.7326/M20-6306 PMC7772883

[25] J. Riou , R. Panczak , C. L. Althaus , et al., “Socioeconomic Position and the COVID‐19 Care Cascade From Testing to Mortality in Switzerland: A Population‐Based Analysis,” The Lancet Public Health 6, no. 9 (2021): e683–e691. 34252364 10.1016/S2468-2667(21)00160-2 PMC8270761

[26] Y. Zhu , Y. Xia , J. Pickering , A. C. Bowen , and K. R. Short , “The Role of Children in Transmission of SARS‐CoV‐2 Variants of Concern Within Households: An Updated Systematic Review and Meta‐Analysis, as at 30 June 2022,” Eurosurveillance 28, no. 18 (2023): 2200624. 37140450 10.2807/1560-7917.ES.2023.28.18.2200624 PMC10161681

[27] R. Naeimi , M. Sepidarkish , A. Mollalo , et al., “SARS‐CoV‐2 Seroprevalence in Children Worldwide: A Systematic Review and Meta‐Analysis,” eClinicalMedicine 56 (2023): 56. 10.1016/j.eclinm.2022.101786 PMC9795163 36590788

[28] M. A. J. Luijten , M. M. van Muilekom , L. Teela , et al., “The Impact of Lockdown During the COVID‐19 Pandemic on Mental and Social Health of Children and Adolescents,” Quality of Life Research 30, no. 10 (2021): 2795–2804. 33991278 10.1007/s11136-021-02861-x PMC8122188

[29] B. de Gier , A. J. Huiberts , C. E. Hoeve , et al., “Effects of COVID‐19 Vaccination and Previous Infection on Omicron SARS‐CoV‐2 Infection and Relation With Serology,” Nature Communications 14, no. 1 (2023): 4793. 10.1038/s41467-023-40195-z PMC10412579 37558656

[30] U. Marking , O. Bladh , S. Havervall , et al., “7‐Month Duration of SARS‐CoV‐2 Mucosal Immunoglobulin‐a Responses and Protection,” The Lancet Infectious Diseases 23, no. 2 (2023): 150–152. 36640796 10.1016/S1473-3099(22)00834-9 PMC9833832

[31] M. K. Verheul , J. Kaczorowska , M. I. Hofstee , et al., “Protective Mucosal SARS‐CoV‐2 Antibodies in the Majority of the General Population in the Netherlands,” Mucosal Immunology. Published ahead of print, March 27, 2024. 10.1016/j.mucimm.2024.03.008. 38553008

[32] N. Bobrovitz , H. Ware , X. Ma , et al., “Protective Effectiveness of Previous SARS‐CoV‐2 Infection and Hybrid Immunity Against the Omicron Variant and Severe Disease: A Systematic Review and Meta‐Regression,” The Lancet Infectious Diseases 23, no. 5 (2023): 556–567. 36681084 10.1016/S1473-3099(22)00801-5 PMC10014083

[33] G. den Hartog , E. R. A. Vos , L. L. van den Hoogen , et al., “Persistence of Antibodies to SARS‐CoV‐2 in Relation to Symptoms in a Nationwide Prospective Study,” Clinical Infectious Diseases 73 (2021): 2155–2162. 33624751 10.1093/cid/ciab172 PMC7929058

[34] G. den Hartog , S. Andeweg , C. Hoeve , et al., “Assessment of Hybrid Population Immunity to SARS‐CoV‐2 Following Breakthrough Infections of Distinct SARS‐CoV‐2 Variants,” Scientific Reports 13 (2023): 18394. 37884642 10.1038/s41598-023-45718-8 PMC10603038

[35] A. M. D. Navaratnam , M. Shrotri , V. Nguyen , et al., “Nucleocapsid and Spike Antibody Responses Following Virologically Confirmed SARS‐CoV‐2 Infection: An Observational Analysis in the Virus Watch Community Cohort,” International Journal of Infectious Diseases 123 (2022): 104–111. 35987470 10.1016/j.ijid.2022.07.053 PMC9385348

[36] J. A. Backer , L. Mollema , E. R. A. Vos , et al., “Impact of Physical Distancing Measures Against COVID‐19 on Contacts and Mixing Patterns: Repeated Cross‐Sectional Surveys, the Netherlands, 2016–17, April 2020 and June 2020,” Eurosurveillance 26, no. 8 (2021): 2000994. 33632374 10.2807/1560-7917.ES.2021.26.8.2000994 PMC7908067

[37] J. A. Backer , L. Bogaardt , P. Beutels , et al., “Dynamics of Non‐Household Contacts During the COVID‐19 Pandemic in 2020 and 2021 in the Netherlands,” Scientific Reports 13, no. 1 (2023): 5166. 36997550 10.1038/s41598-023-32031-7 PMC10060924

[38] G. den Hartog , P. B. van Kasteren , R. M. Schepp , A. C. Teirlinck , F. R. M. van der Klis , and R. S. van Binnendijk , “Decline of RSV‐Specific Antibodies During the COVID‐19 Pandemic,” The Lancet Infectious Diseases 23, no. 1 (2023): 23–25. 36463892 10.1016/S1473-3099(22)00763-0 PMC9714975

[39] B. de Gier , N. Marchal , I. de Beer‐Schuurman , et al., “Increase in Invasive Group a Streptococcal (Streptococcus Pyogenes) Infections (iGAS) in Young Children in the Netherlands, 2022,” Eurosurveillance. 28, no. 1 (2023): 2200941. 36695447 10.2807/1560-7917.ES.2023.28.1.2200941 PMC9817208

