# Supplementary material for: SARS‐CoV‐2 Seroprevalence Trends in the Netherlands in the Variant of Concern Era: Input for Future Response
Source: Influenza Other Respir Viruses. 2024 Jun 4;18(6):e13312. doi: 10.1111/irv.13312 (PMC11150416; doi:10.1111/irv.13312)
Supplement: Supplementary file 1 — Table S1 Overview of responders vs. non‐responders from the additional sampling in Nov. 2021 (PICO6). Figure S1. Number of participants in the current PIENTER Corona (PICO) study belonging to the national sample and low vaccination coverage (LVC) sample (as conceived in the PIENTER‐3 cohort) in 2021 (February (PICO4), June (PICO5) and November (PICO6)) and 2022 (March (PICO7), June (PICO8), November (PICO9)), by age categories (years) and region. Regions consist of the following provinces:North = Groningen, Friesland, Drenthe and Overijssel; Mid‐West = Flevoland and Noord‐Holland; Mid‐East = Gelderland and Utrecht; South‐West = Zuid‐Holland and Zeeland; and South‐East = Noord‐Brabant and Limburg. Figure S2. Weighted vaccination coverage (%) in the general Dutch population (as derived from the national sample of the current PIENTER Corona (PICO) study cohort) in 2021 (February (PICO4), June (PICO5) and November (PICO6)) and 2022 (March (PICO7), June (PICO8), November (PICO9)), by sex, age categories (years) and number of vaccine doses. The vaccination campaign roll‐out followed an age‐, comorbidity‐, and healthcare worker‐based prioritization that started in the beginning of 2021. Figure S3. Weighted SARS‐CoV‐2 seroprevalence induced by infection (a) and total (i.e., infection and vaccination) (b) in the general Dutch population in 2021 (February (PICO4), June (PICO5) and November (PICO6)) and 2022 (March (PICO7), June (PICO8), November (PICO9)), by Municipality Health Service (GGD) region. Figure S4. Weighted SARS‐CoV‐2 seroprevalence (with 95% confidence intervals) induced by infection (a) and total (i.e., infection and vaccination) (b) in the general Dutch population in 2021 (February (PICO4), June (PICO5) and November (PICO6)) and 2022 (March (PICO7), June (PICO8), November (PICO9)), by age categories (years) and sex. Figure S5. Weighted SARS‐CoV‐2 seroprevalence (with 95% confidence intervals) induced by infection (a) and total (i.e., infection and vaccination) [file IRV-18-e13312-s001.pdf]

SARS-CoV-2 Seroprevalence Trends in the Netherlands in the Variant of Concern Era: Input for Future Response

Eric R.A. Vos, Cheyenne C.E. van Hagen, Denise Wong, Gaby Smits, Marjan Kuijer, Alienke J. Wijmenga-Monsuur, Joanna Kaczorowska, Robert S. van Binnendijk, Fiona R.M. van der Klis, Gerco den Hartog, Hester E. de Melker

Supplementary material

Supplementary information: additional sampling of the national sample, November 2021 (PICO6)

Details on previous sampling up to round 2 of the PICO-study and sample size calculation have been described in Vos *et al.* (2021, JECH) and Vos *et al*. (2021, CID). Due to drop outs in study rounds after PICO2 - particularly notable in younger age groups - and hence to maintain power for subsequent rounds, the Dutch cohort was supplemented with an additional sample of randomly-selected persons from the Dutch population registry (as of October 1st, 2021) prior to the start of the 6th study round (November 2021). Congruent with the previous study designs, invitees were randomly drawn from five large regions with roughly the same population size (consisting of provinces: North=Groningen, Friesland, Drenthe and Overijssel; Mid-West=Flevoland and Noord-Holland; Mid-East=Gelderland and Utrecht; South-West=Zuid-Holland and Zeeland; and South-East=Noord-Brabant and Limburg), and from 17 pre-defined age groups (1–4, 5–9, 10–14, 15–19, 20–24, 25–29, 30–34, 35–39, 40–44, 45–49, 50–54, 55–59, 60–64, 65–69, 70–74, 75–79, 80–89 years). A minimum of 400 participants per age stratum was anticipated, taking into account initial response rates as well as drop-out rates per age stratum during the study. Taken together, for the additional sample we randomly selected 66,020 persons, of which 65,690 remained eligible for invitation after initial screening, and of these 3,181 participated. The national sample is further described per study round (4 to 9) in 2021 and 2021 and other relevant characteristics in Table 1 of the main article. Supplementary Table S1 below provides an overview of responders vs. non-responders from the additional sampling, stratified by available sociodemographic variables.

Supplementary Table S1. Overview of responders vs. non-responders from the additional sampling in Nov. 2021 (PICO6).

		**Non-responder**		**Responder**		**Total invited additional sample**
		n=62,509 (95.2%)		*n*=3,181 (4.8%)		*n*=65,690
		n	*%*	*n*	*%*	*n*
**Sex**	Man	30,967	49.5	1,364	42.9	32,331
	Women	31,542	50.5	1,817	57.1	33,359
**Age categories (years)**	1–4	18,948	30.3	194	6.1	19,142
	5–9	7,802	12.5	262	8.2	8,064
	10–14	4,275	6.8	218	6.9	4,493
	15–19	3,228	5.2	132	4.1	3,360
	20–24	4,961	7.9	211	6.6	5,172
	25–29	3,557	5.7	184	5.8	3,741
	30–34	1,560	2.5	120	3.8	1,680
	35–39	2,392	3.8	226	7.1	2,618
	40–44	1,107	1.8	136	4.3	1,243
	45–49	1,405	2.3	164	5.2	1,569
	50–54	769	1.2	122	3.8	891
	55–59	502	0.8	98	3.1	600
	60–64	454	0.7	90	2.8	544
	65–69	201	0.3	53	1.7	254
	70–74	870	1.4	186	5.8	1,056
	75–79	2,492	4.0	377	11.9	2,869
	80–84	2,481	4.0	201	6.3	2,682
	85+	5,505	8.8	207	6.5	5,712

**Region**	North	12,492	20.0	655	20.6	13,147
	Mid-West	12,527	20.0	599	18.8	13,126
	Mid-East	12,466	19.9	667	21.0	13,133
	South-West	12,537	20.1	600	18.9	13,137
	South-East	12,485	20.0	660	20.7	13,145
**Urbanization degree**	High (large cities)	15,536	24.9	672	21.1	16,208
	Middle (moderate cities)	19,099	30.5	991	31.2	20,090
	Low (villages to countryside)	27,872	44.6	1,518	47.7	29,390
**Country of birth**	Dutch	56,692	90.7	3,001	94.3	59,693
	Non-Dutch	5,817	9.3	180	5.7	5,997

Ethnic background was not available for non-responders, instead country of birth was used for comparison with responders. Missing: *n*=2 for region and urbanization degree in non-responders. Regions consist of the following provinces: North=Groningen, Friesland, Drenthe and Overijssel; Mid-West=Flevoland and Noord-Holland; Mid-East=Gelderland and Utrecht; South-West=Zuid-Holland and Zeeland; and South-East=Noord-Brabant and Limburg.

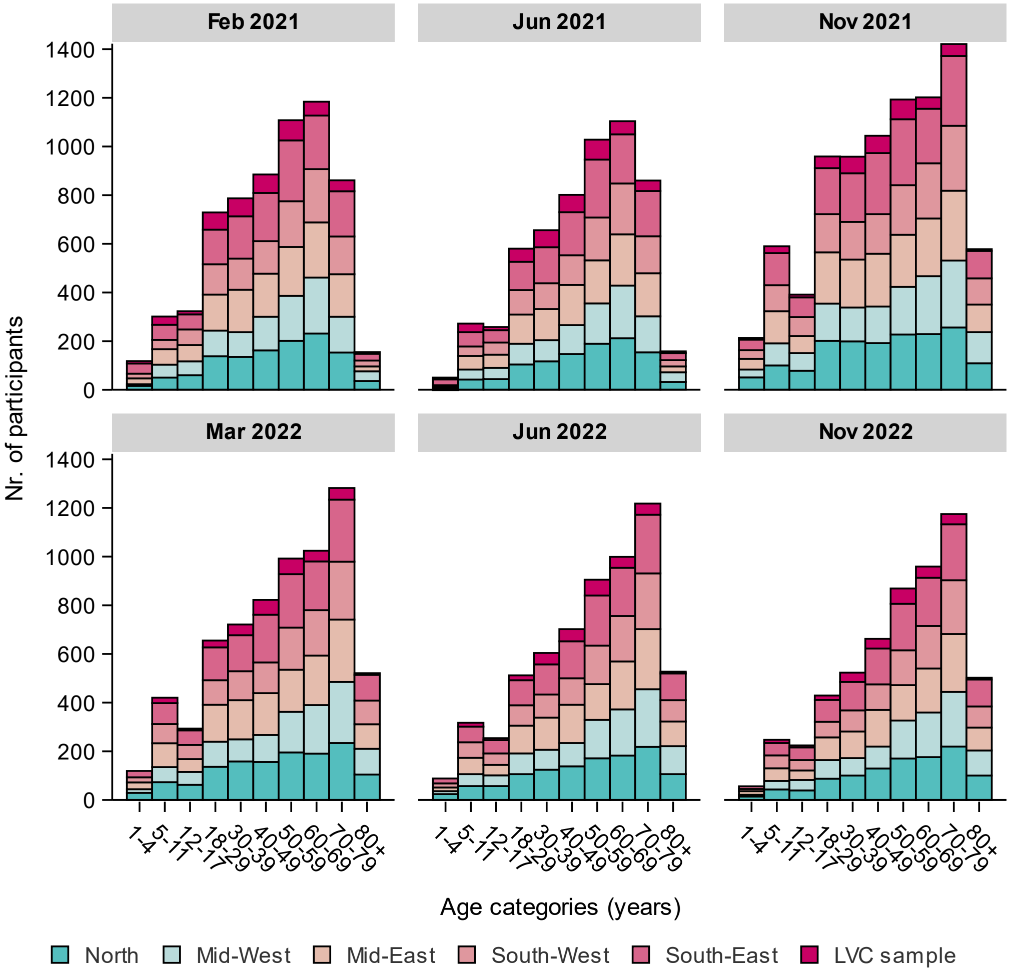

Supplementary Figure S1. Number of participants in the current PIENTER Corona (PICO) study belonging to the national sample and low vaccination coverage (LVC) sample (as conceived in the PIENTER-3 cohort) in 2021 (February (PICO4), June (PICO5) and November (PICO6)) and 2022 (March (PICO7), June (PICO8), November (PICO9)), by age categories (years) and region. Regions consist of the following provinces: North=Groningen, Friesland, Drenthe and Overijssel; Mid-West=Flevoland and Noord-Holland; Mid-East=Gelderland and Utrecht; South-West=Zuid-Holland and Zeeland; and South-East=Noord-Brabant and Limburg.

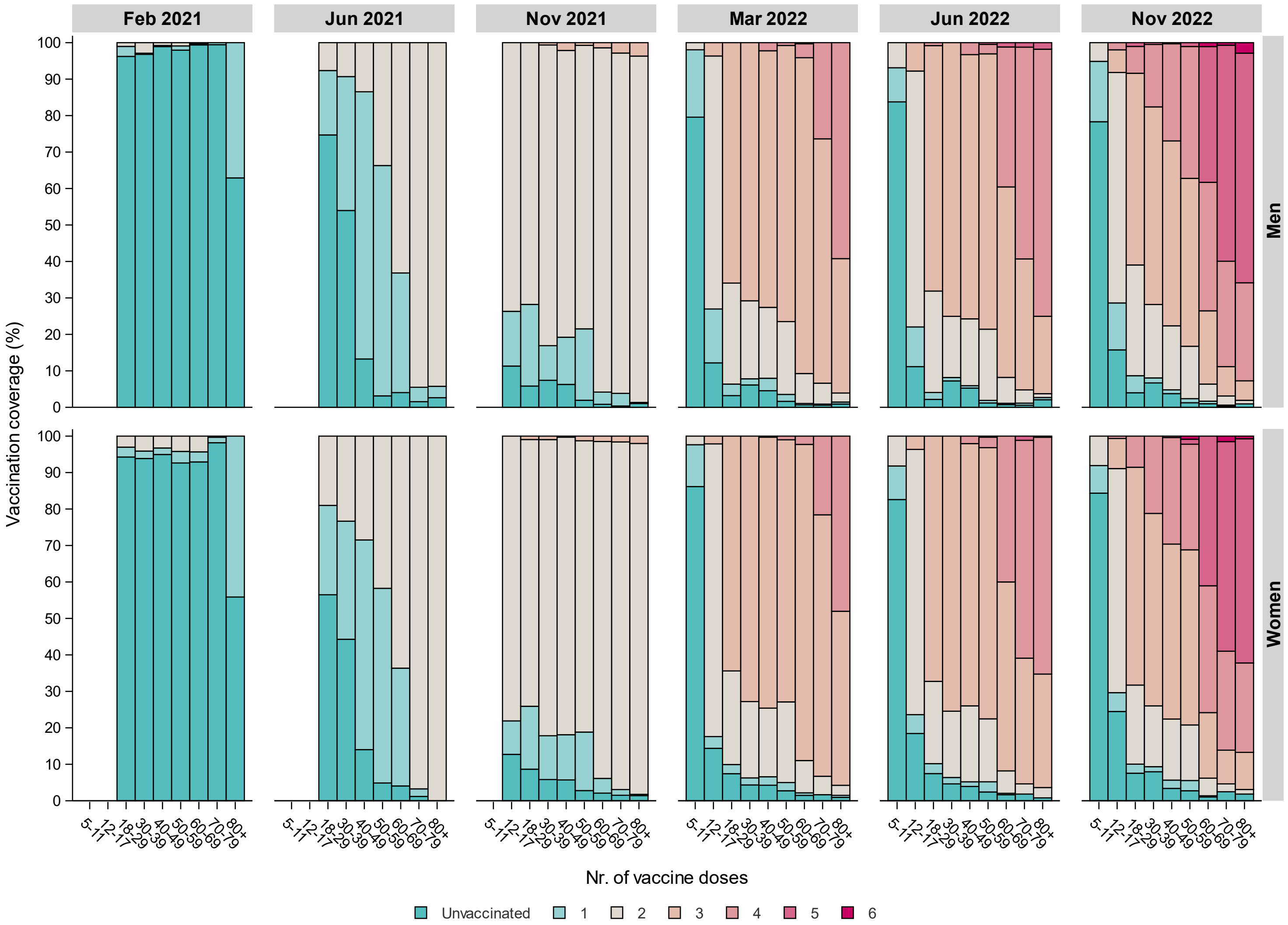

Supplementary Figure S2. Weighted vaccination coverage (%) in the general Dutch population (as derived from the national sample of the current PIENTER Corona (PICO) study cohort) in 2021 (February (PICO4), June (PICO5) and November (PICO6)) and 2022 (March (PICO7), June (PICO8), November (PICO9)), by sex, age categories (years) and number of vaccine doses. The vaccination campaign roll-out followed an age-, comorbidity-, and healthcare worker-based prioritization that started in the beginning of 2021.

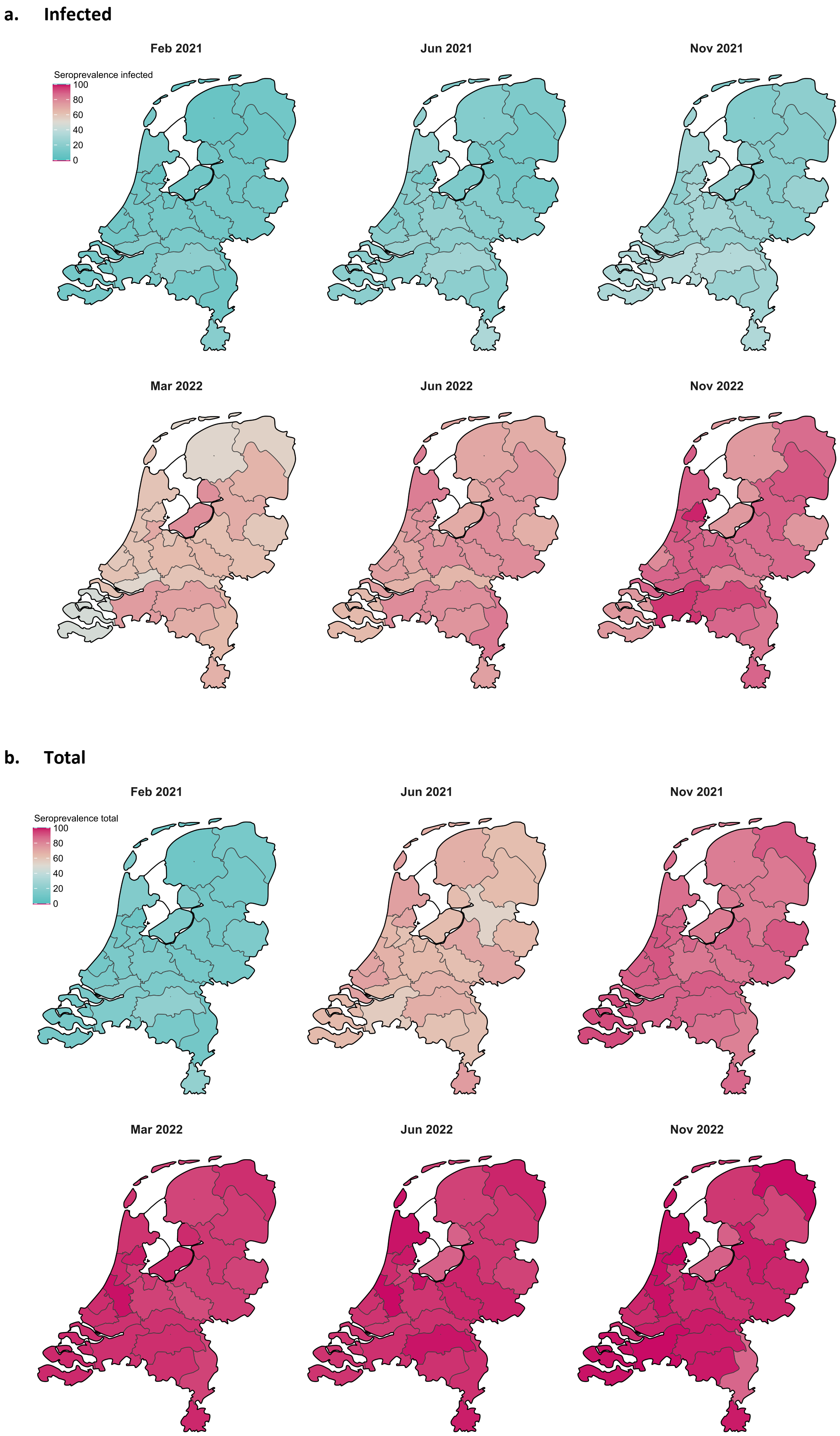

Supplementary Figure S3. Weighted SARS-CoV-2 seroprevalence induced by infection (**a**) and total (i.e., infection and vaccination) (**b**) in the general Dutch population in 2021 (February (PICO4), June (PICO5) and November (PICO6)) and 2022 (March (PICO7), June (PICO8), November (PICO9)), by Municipality Health Service (GGD) region.

a. Infected

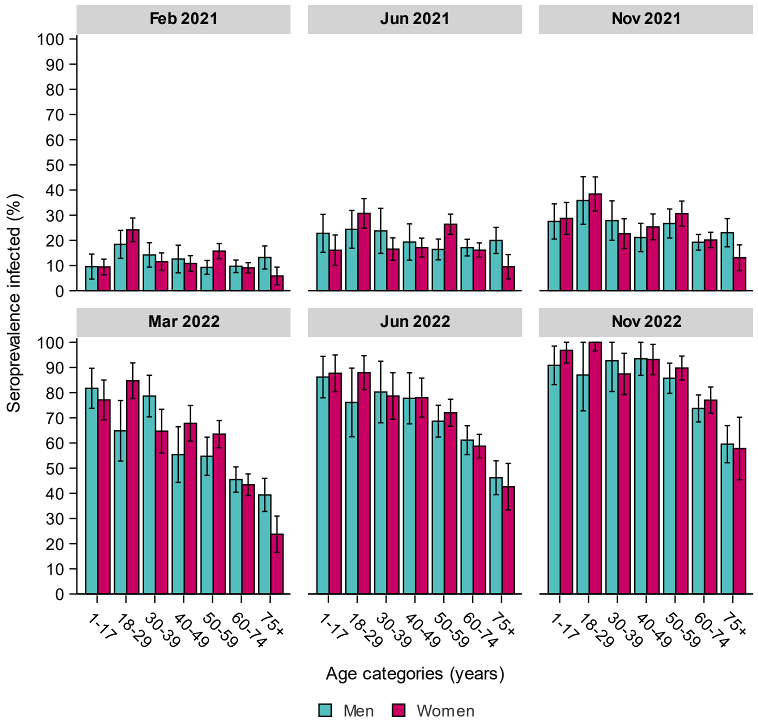

b. Total

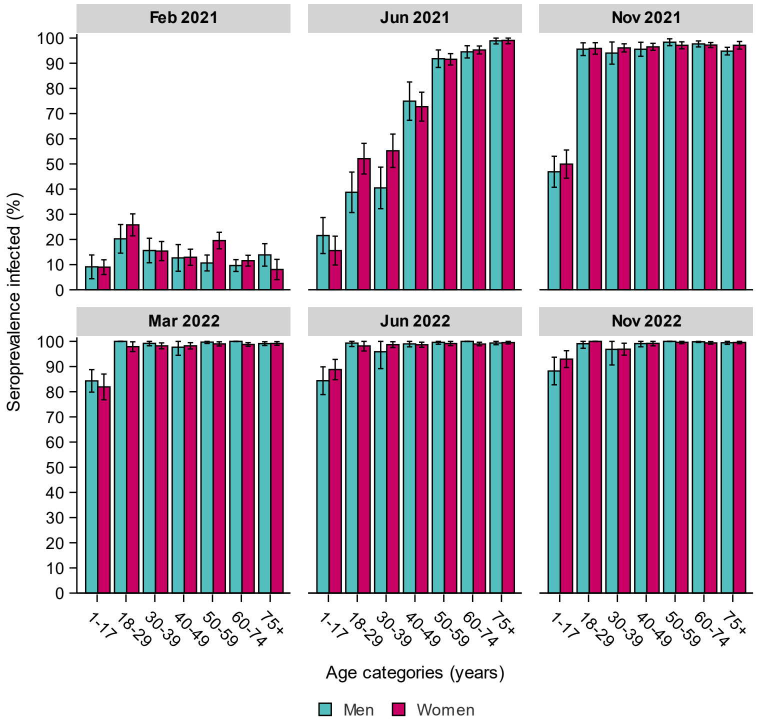

Supplementary Figure S4. Weighted SARS-CoV-2 seroprevalence (with 95% confidence intervals) induced by infection (**a**) and total (i.e., infection and vaccination) (**b**) in the general Dutch population in 2021 (February (PICO4), June (PICO5) and November (PICO6)) and 2022 (March (PICO7), June (PICO8), November (PICO9)), by age categories (years) and sex.

a. Infected

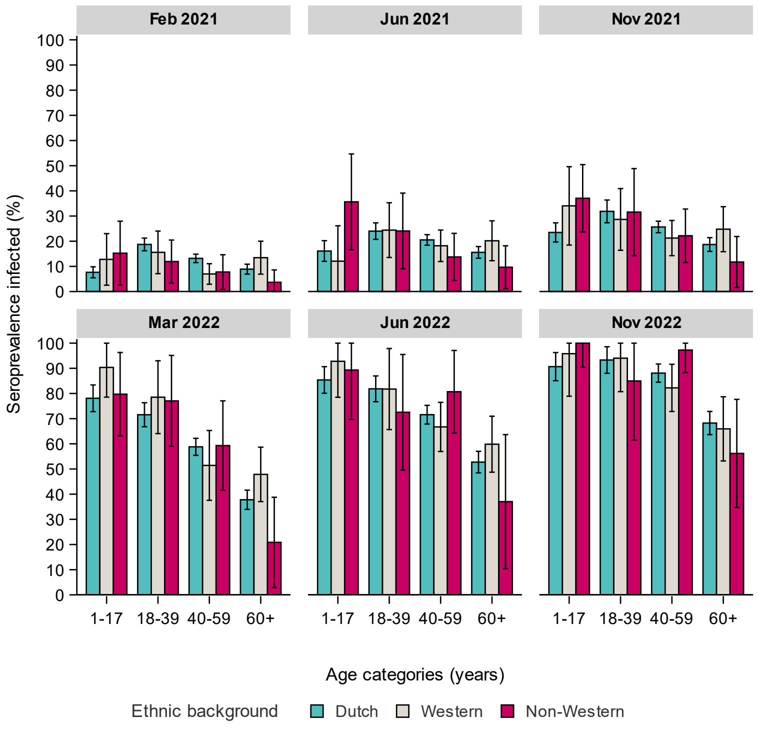

b. Total

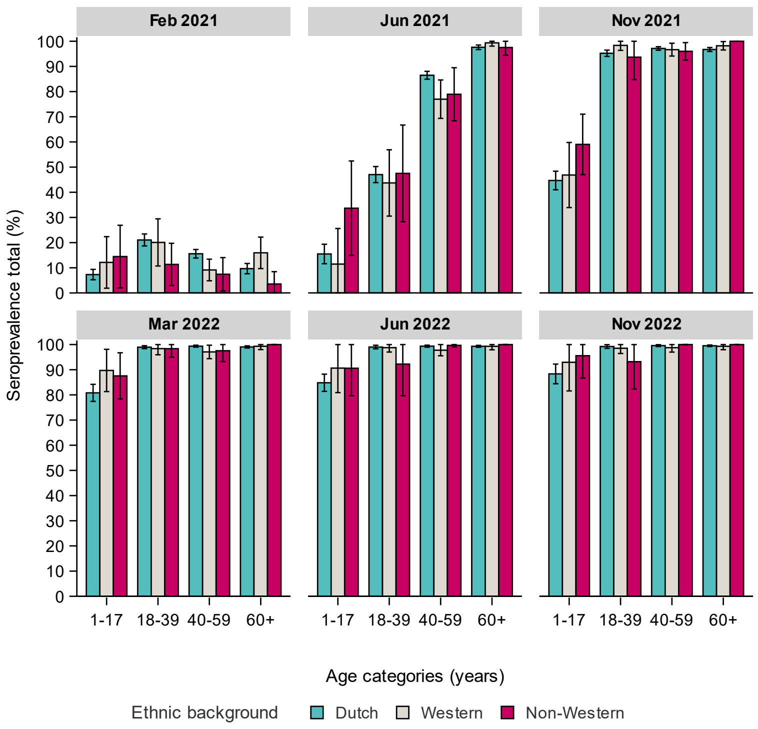

Supplementary Figure S5. Weighted SARS-CoV-2 seroprevalence (with 95% confidence intervals) induced by infection (**a**) and total (i.e., infection and vaccination) (**b**) in the general Dutch population in 2021 (February (PICO4), June (PICO5) and November (PICO6)) and 2022 (March (PICO7), June (PICO8), November (PICO9)), by age categories (years) and ethnic background.

a. Infected

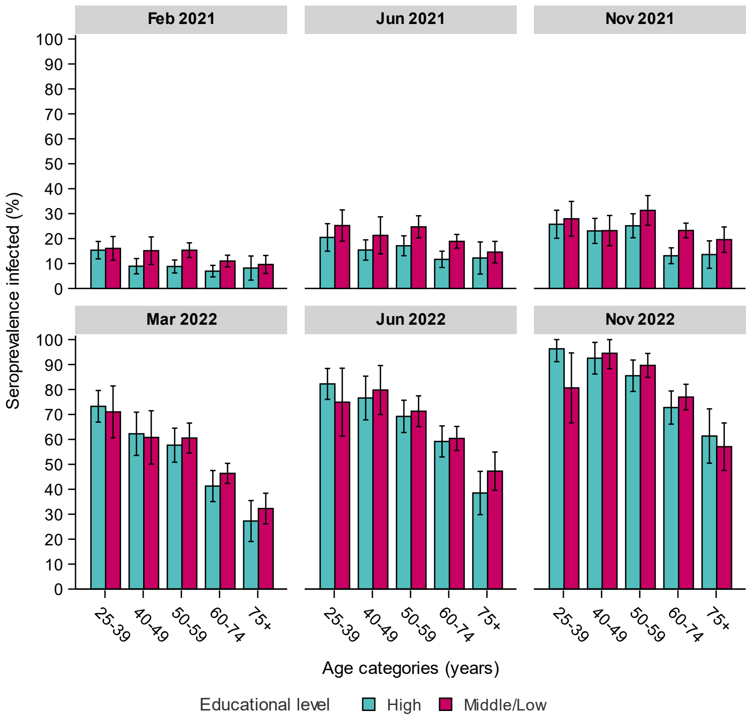

b. Total

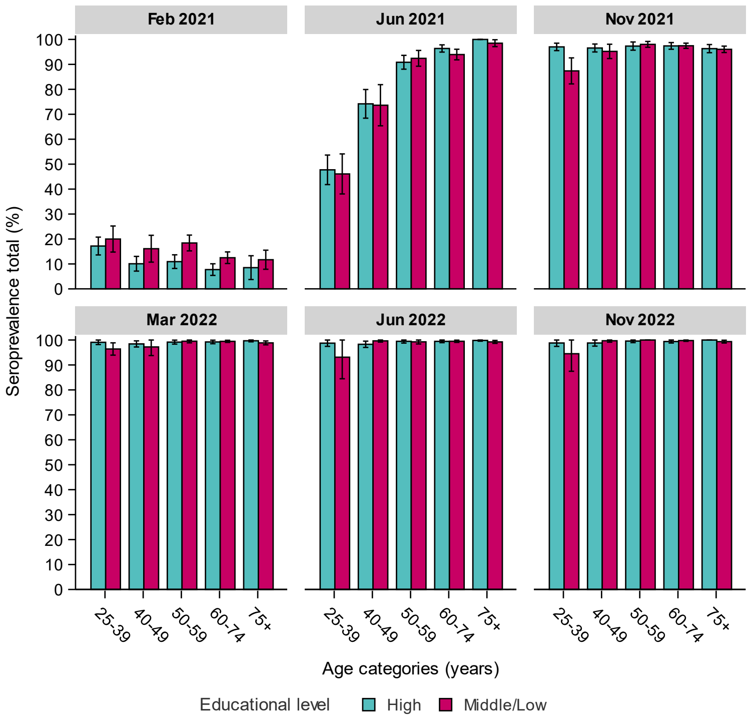

Supplementary Figure S6. Weighted SARS-CoV-2 seroprevalence (with 95% confidence intervals) induced by infection (**a**) and total (i.e., infection and vaccination) (**b**) in the general Dutch population in 2021 (February (PICO4), June (PICO5) and November (PICO6)) and 2022 (March (PICO7), June (PICO8), November (PICO9)), by age categories (years) and educational level ((from 25 years of age, highest obtained or current). Educational level was classified as low (no education or primary education)/middle (secondary school or vocational training), or high (bachelor's degree, university)).
